# Continental weathering-phosphogenesis coupling across the ediacaran-cambrian transition, Southwestern China

**DOI:** 10.1371/journal.pone.0356723

**Published:** 2026-08-27

**Authors:** Yuchuan Yang, Qiang Hao, Jun Wen, Wei Zhao, Chi Zhang

**Affiliations:** 1 The 7th Geological Brigade of Sichuan, Leshan, China; 2 The Engineering & technical College of Chengdu University of Technology, Leshan, China; 3 State Key Laboratory of Marine Geology, Tongji University, Shanghai, China; 4 Institute of Exploration and Development, Petro China Southwest Oil and Gasfield Company, Chengdu, China; Birbal Sahni Institute of Palaeosciences: Birbal Sahni Institute of Palaeobotany, INDIA

## Abstract

Phosphorus, as a primary limiting element for ecosystem productivity, plays a pivotal role in regulating the redox state of the atmosphere-ocean system and climate change. Under-standing the coupling relationship between the phosphorus cycle and climate dynamics during geological history has long been a research challenge in Earth system science. Here we are primarily based on sedimentary profiles and rock samples from phosphate mining areas on the western margin of the Yangtze Plate. By analyzing multiple elemental geo-chemical proxies, it reconstructs the depositional environment and phosphorus enrichment mechanisms during the Meishucun Age of the Early Cambrian. Our results show that: (1) The total phosphorus flux from continental weathering is jointly controlled by tectonic activity and climate change. The intensity of tectonic activity affects the rate of rock erosion, which determines the total phosphorus flux, while climate influences the in-tensity of chemical weathering, which controls the proportion of reactive phosphorus; (2) During the Ediacaran-Cambrian (E-Ꞓ) transition, due to the continuous increase in atmospheric oxygen concentration, the Yangtze region experienced intense chemical weathering. Concomitant with the occurrence of a “Great Unconformity” at the Ediacaran/Cambrian boundary, weathering of continental (phosphorus-bearing) rocks constituted the primary source of phosphorus in the oceans; and (3) Upwelling transported particulate and dissolved phosphorus from the marine phosphorus reservoir to shallow-water environments, where physical concentration, biological processes and potential hydrothermal activity contributed to phosphorus enrichment to different extents, ultimately leading to the formation of high-grade phosphorites. Collectively, these findings highlight the complexity of marine phosphorus enrichment mechanisms in the Early Cambrian and provide additional constraints for understanding the coupling relationship between phosphorus burial and organic carbon burial.

## 1. Introduction

Phosphorus is a key nutrient essential for life on Earth. It is a major component of DNA (deoxyribonucleic acid) and RNA (ribonucleic acid), and, through the synthesis of ATP (adenosine triphosphate), it participates in energy transfer, thereby playing a role in cellular metabolism [1,2]. Recent studies have shown that the source-sink balance of phosphorus in the global oceans directly constrains the level of primary productivity and controls the rate of photosynthesis, which in turn directly affects the global carbon-oxygen cycle, marine redox state, climate change, and even biological evolution processes [1–4]. In marine systems, phosphorus is buried in sediments mainly through two pathways: terrigenous input of detrital phosphorus and removal of recycled phosphorus from water bodies (organic matter mineralization and iron oxides binding) [3,5]. Accordingly, reconstructing temporal variations in phosphorus contents of sedimentary rocks during different geological period is of critical insights for understanding the level of biological productivity, the coupling relationship between the C-O-P cycles, and the long-term feedback mechanisms of climate change.

The Ediacaran-Cambrian transition is a pivotal period in Earth’s evolution, during which a series of global geological events occurred successively, including the Snowball Earth events, the breakup of the Rodinia supercontinent, global anoxia, sea-level rise, and the Cambrian Explosion. These events have exerted a profound impact on the evolution of Earth’s environments and life [6,7]. In many global tectonic plates, the Neoproterozoic and Phanerozoic strata are separated by the “Great Unconformity,” which is regarded as a critical carrier for studying key scientific issues such as global climate change and the Cambrian Explosion. Recent studies have shown that the significant increase in marine alkalinity and the intensity of continental weathering and erosion exhibit a strong correlation with environmental changes, biological evolution, and phosphorus-forming events [6–9]. As a major constituent of the Gondwanan paleocontinent, the Yangtze Plate has a direct connection with the breakup of Rodinia supercontinent and the assembly of Gondwana. It is thus an ideal site for studying the tectono-sedimentary evolution, phosphorus-forming events, and climate changes during the Neoproterozoic to Early Cambrian periods.

As a major depocenter of sediments during the E-Ꞓ transition, the Yangtze Plate is an ideal archive for reconstructing the depositional environment and phosphorus enrichment mechanisms. This study focuses on the Tongchanggeng (TCG) and Huangjiaping (HJP) sections in the Yangtze Block and, by integrating available sedimentary geochemical datasets, attempts to (1) analyze the contact relationship between the Ediacaran and Cambrian strata on the western margin of the Yangtze Plate; (2) evaluate the redox conditions of the ocean; and (3) provide additional constraints for understanding the mechanisms of phosphorus enrichment and climate change during the Early Cambrian.

## 2. Geological setting

**Fig 1:**
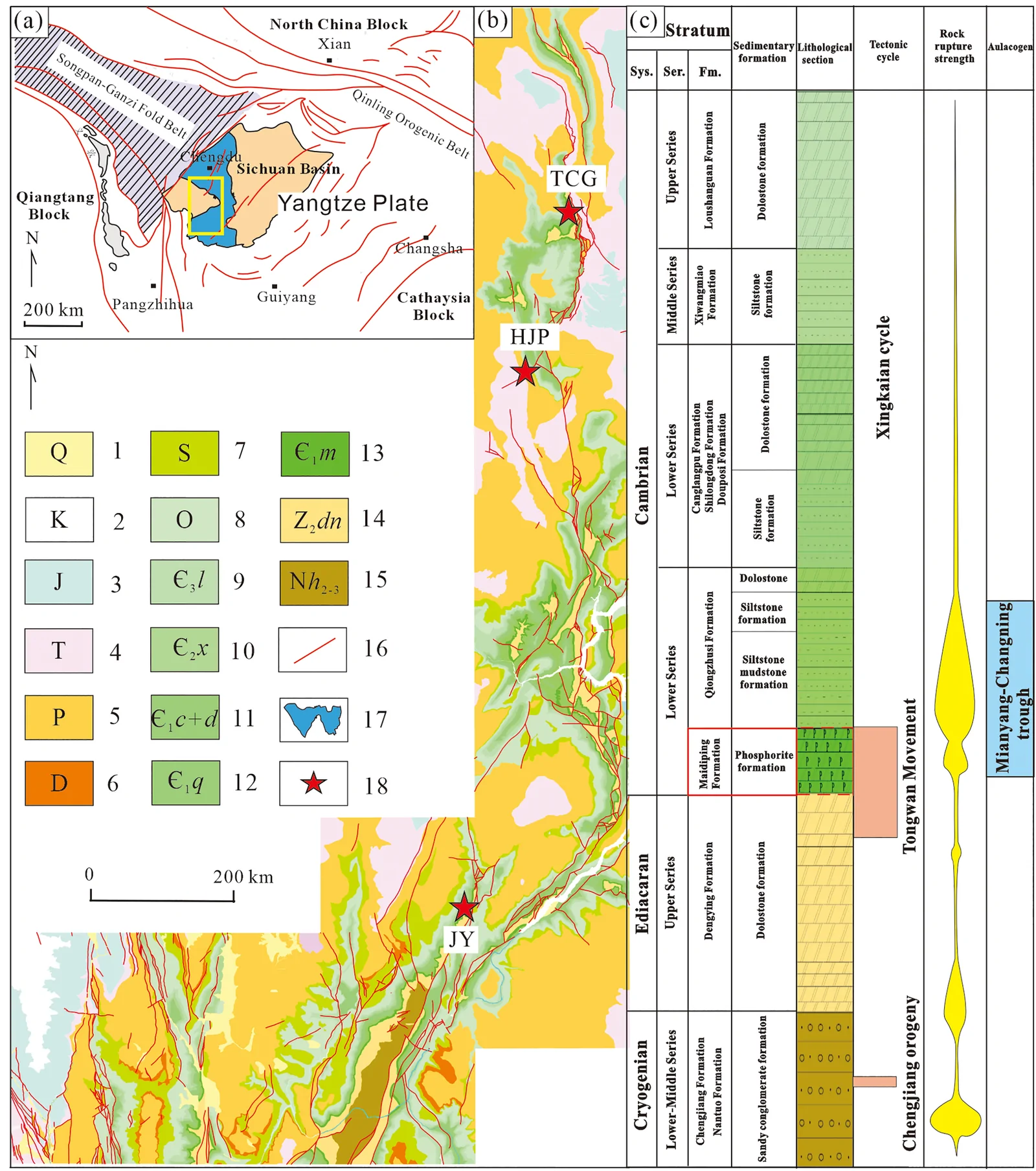
Geological structural background of the study area. (a) Tectonic map of the Sichuan Basin peripheral region [6]; (b) Geological map of the study area and locations of sections; (c) Composite stratigraphic column of the Cambrian strata in the study area. 1. Quaternary; 2. Cretaceous; 3. Jurassic; 4. Triassic; 5. Permian; 6. Devonian; 7. Silurian; 8. Ordovician; 9. Loushanguan Formation; 10. Xiwangmiao Formation; 11. Canglangpu/Douposi Formation; 12. Qiongzhusi Formation; 13. Maidiping Formation; 14. Dengying Formation; 15. Nanhua System; 16. fault; 17. extensional trough; 18. section;.

The crystalline basement of the Yangtze Platform was formed approximately 1700 Ma ago during the Paleoproterozoic [10,11]. Following the Jinning Movement, the overall framework of the ancient Yangtze Plate was established (Fig 1). While traditional views hold that the plate attained a stable cratonic nature after entering the Caledonian Period [12–14], more recent studies demonstrate that it remained tectonically active, experiencing intense extensional deformation during the Ediacaran-Cambrian transition [15,16], accompanied by multiple phases of marine transgression and regression (Fig 1c). During this period, the Yangtze Plate evolved from a rift basin into a passive continental margin basin, flanked by the Qinling Sea to the northwest and the Nanhua Ocean to the southeast, and the overall topography featured a “higher west and lower east” pattern [17]. Paleogeographic reconstructions integrating detrital zircon U-Pb ages, carbon isotope records, and paleontological patterns suggest that by the Early Cambrian, the Yangtze Plate was well connected to the open ocean. Upwelling currents carrying large quantities of nutrients invaded the region, leading to extensive deposition of carbonate rocks, phosphatic rocks, and organic-rich shales [18]. Specifically, the shelf facies was dominated by carbonate and phosphatic rock deposits; the depressed basins within the platform troughs were characterized by mixed deposits of siliceous rocks, shales, carbonates, and phosphatic rocks; the slope facies (transition zone) was well-developed with fossil-rich black shales; and the deep-water areas were mainly composed of siliceous rocks with few fossils [19].

**Fig 2:**
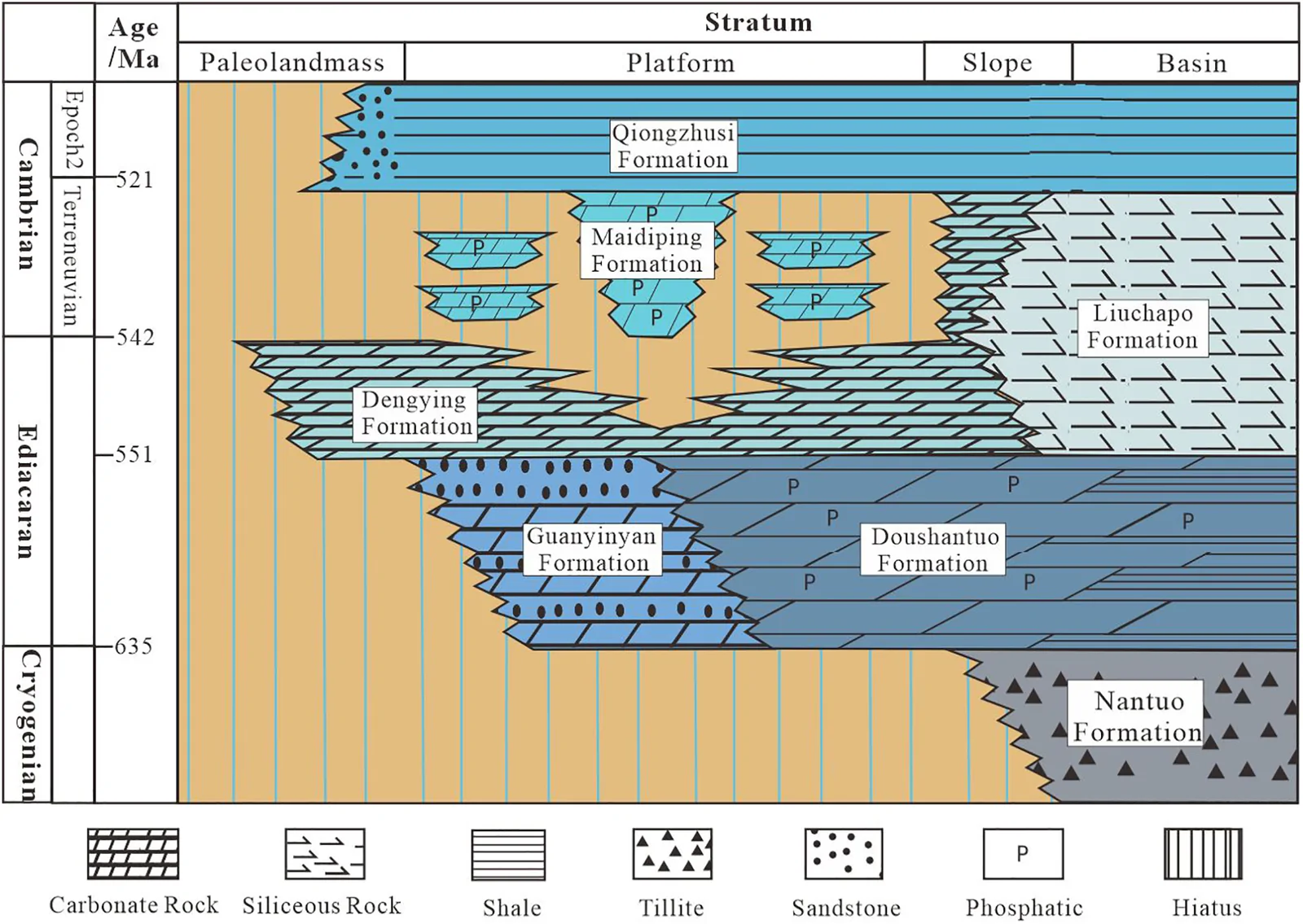
Lithostratigraphic characteristics of the Upper Cryogenian to Lower Cambrian on the western margin of the Yangtze Plate.

In terms of stratigraphy, intense extensional activities and sea-level fluctuations collectively controlled the lithological and stratigraphic architecture of the Yangtze region from the late Cryogenian to the Early Cambrian [6,7,9,10], (Fig 1c and 2). The upper part of the Cryogenian System was dominated by the Nantuo Formation, which was mainly developed in rift basins and composed primarily of tillite. With the end of the Cryogenian, sea levels rose, leading to extensive deposition of carbonate rocks and phosphatic rocks of the Ediacaran Doushantuo Formation; in areas close to the ancient continent, this evolved into the Guanyinya Formation, which contained clastic rocks [11–16]. By the late Ediacaran, carbonate platforms further expanded, and micritic, clastic, and algal dolomites were well-developed, forming the Dengying Formation(Z_2_dn) In some local areas, the carbonate rocks of the Dengying Formation transitioned facies into the siliceous rocks of the Liuchapo Formation [17–19]. During the deposition from the Ediacaran Dengying Formation to the Early Cambrian Maidiping Formation(Ꞓ_1_m), affected by multiple phases of the Tongwan Movement, the strata often showed varying degrees of absence. The Maidiping Formation was notably thin in both the northern and southern Yangtze Plate and had an unconformable contact with the underlying Dengying Formation [14]. There were two types of contact relationships between the Qiongzhusi Formation exhibits and the underlying Maidiping Formation: conformable contact and disconformable (parallel unconformable) contact. In some local areas, the Qiongzhusi Formation directly onlapped the Dengying Formation, with the Maidiping Formation missing [6,10]. Notably, during the depositional period of the Maidiping Formation, a tidal flat-shoal-coastal sedimentary system developed on the western margin of the Yangtze Plate. Among this system, the tidal-flat and subtidal-bay facies belts were the main enrichment areas for phosphorites [20], collectively forming China’s major phosphorite metallogenic belt and representing a key metallogenic period.

## 3. Sample materials and analytical methods

**Fig 3:**
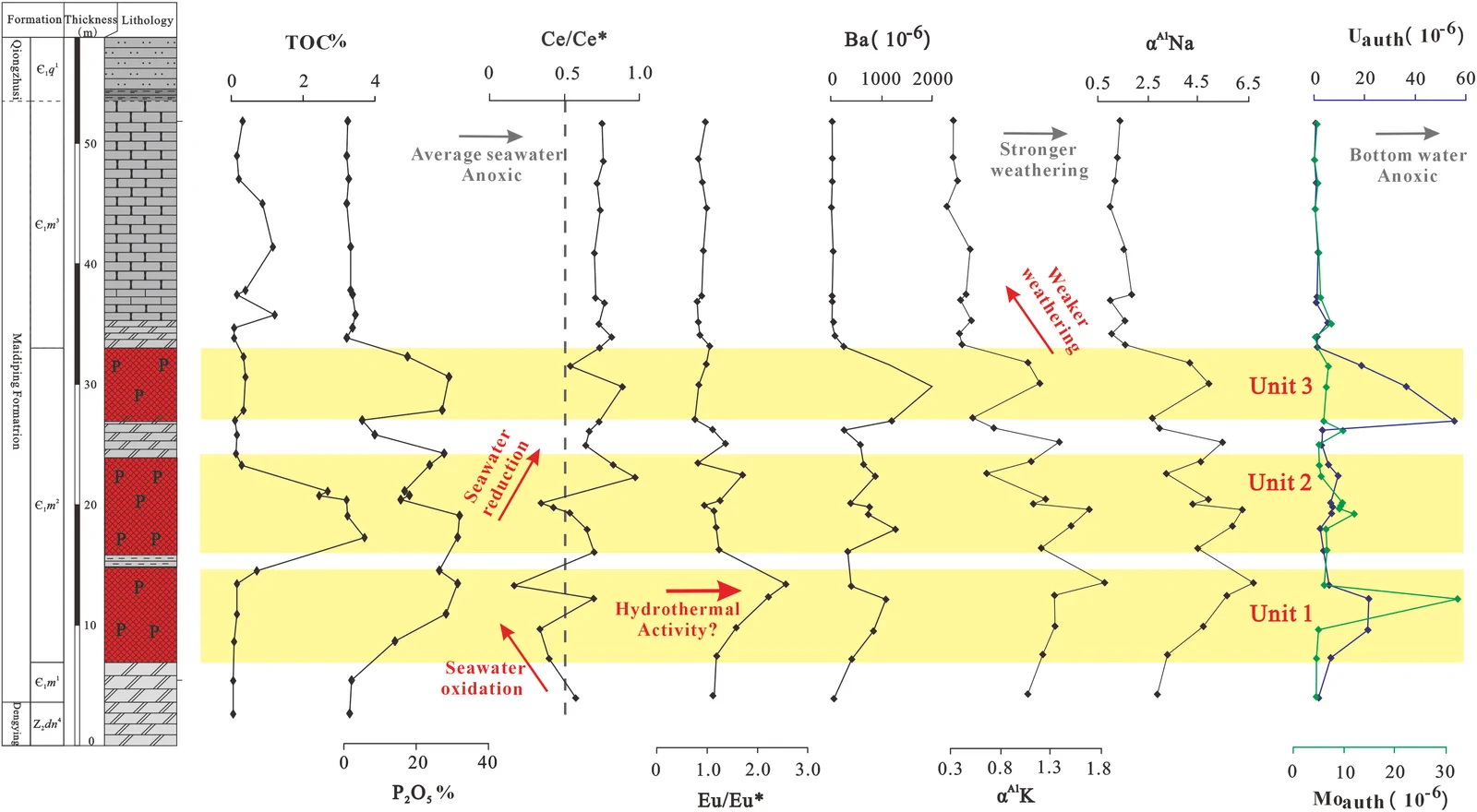
Downcore variations in TOC, P_2_O_5_, Ba_auth_, U_auth_, Mo_auth_ Contents, α^Al^Na, α^Al^K, Ce/Ce* and Eu/Eu* ratios for the sediments of the TCG section.

No specific permits were required for field sampling because all samples were collected from publicly accessible outcrops and no protected areas or endangered species were involved. The TCG and HJP sections, located along the western margin of the Yangtze Plate, were investigated and sampled by the China Geological Survey during the implementation of the 1:50,000 Geological and Mineral Survey Project in the peripheral region of the Sichuan Basin [21]. In this study, a total of 38 samples were col-lected and subjected to geochemical analyses of total organic carbon (TOC), major elements, and rare earth elements (REEs), respectively. The sampling locations are shown in Fig 3. All analyses were performed at the Southwest Metallurgical Testing Center, with data provided in the Supplementary Materials S1–S3 Tables.

After grinding the samples to 200 mesh, ~ 100 mg of the ground samples were digested with 5 mL of 50% HNO_3_ and 7 mL of HF. After evaporation to dryness, the residues were diluted to 10 mL with 2% HNO_3_. Major and trace element concentrations were measured using an ICP-MS (Agilent 7900). During the test, a shale standard reference (GBW03014) solution was inserted for precision control, and the test results showed an error less than 5%. Rare earth element (REE) concentrations were normalized using the post-Archean Australian average shale (PAAS) [22]. For TOC measurements, 5–10 g of ground sample was weighed and placed in a 100 mL beaker and treated with an excess of 50% ultrapure HCl to completely remove carbonates from the sample, with intermittent shaking to ensure reaction completion. The insoluble residues were separated by centrifugation, repeatedly washed with ultrapure water until neutral, dried at ~70°C, and subsequently analyzed using a Vario EL Cube elemental analyzer. The geochemical proxies and their corresponding equations are summarized in Table 1.

**Table 1:** Summary of geochemical proxies for chemical weathering and redox Conditions.

Geochemical Proxies	Formula	Reference
α^Al^E	(Al/E)_sample_/(Al/E)_UCC_	[23]
X_auth_	X_auth_ = X_sample_-Al_sample_×(X/Al)_PASS_	[24]
Ce/Ce*	3Ce_PAAS_/(2La_PAAS_+Nd_PAAS_)	[25]
Eu/Eu*	2Eu_PAAS_/(Sm_PAAS_+ Gd_PAAS_)	[25]
Pr/Pr*	Pr/Pr* = 2Pr_PAAS_/(Ce_PAAS_+ Nd_PAAS_)	[25]

Note: The subscript auth indicates the authigenic fraction, E denotes the mobile element, and X denotes U, Mo, or Ba.

## 4. Results

The TOC content in the TCG section is overall low but exhibits considerable variability. The ore-bearing bed in the middle part has relatively high TOC content (2.7%−3.7%; Fig 3), comparable to that of the Cretaceous- Tertiary (C/T) boundary black shales deposited under high-productivity and anoxic conditions [26] (with an average TOC content of 4.1%). The phosphorus content, expressed as P_2_O_5_, is tightly constrained by the phosphorite layers: ranging from 14.2% to 32.0% in the lower layer, 14.4% to 31.3% in the middle layer, and 17.5% to 29.1% in the upper layer, all significantly higher than those of the dolostones and nodular limestones hosting the ore. Ba content peaks in the upper ore-bearing layer, with an average value of 1438.24 × 10^−6^, whereas the middle and lower layers yield average values of 702.62 × 10^−6^ (Fig 3). Correspondingly, elevated α^Al^K and α^Al^Na values characterize the upper Dengying Formation and Members 1–2 of the Maidiping Formation, with mean values of 1.2 and 4.4, respectively, before declining sharply to 0.4 and 1.0 in Member 3. Both indices exhibit a trend comparable to that of P_2_O_5_ concentrations.

In terms of REEs, most samples from the TCG section exhibit negative Ce anomalies (Ce/Ce* < 1; Fig 3), whereas the average Eu/Eu* ratio is greater than 1. A marked positive Eu anomaly is especially evident in the lower ore-bearing layer (Fig 3). The authigenic Uranium (U_auth_), authigenic Molybdenum (Mo_auth_), and authigenic Mo-to-U ratio [(Mo/U)_auth_] are summarized in S3 Table. In general, the contents of U_auth_ and Mo_auth_ in both the ore layers and host rocks of this deposit are relatively low, except for the upper ore layer, where U_auth_ exceeds that of C/T boundary black shales. Moreover, most (Mo/U)_auth_ ratios fall below 1, lower than the corresponding value for black shales (12.25).

## 5. Discussion

### 5.1. Controls on marine phosphorus reservoirs during the E-Ꞓ transition

#### 5.1.1. Marine phosphorus reservoirs and phosphorus cycle.

**Fig 4:**
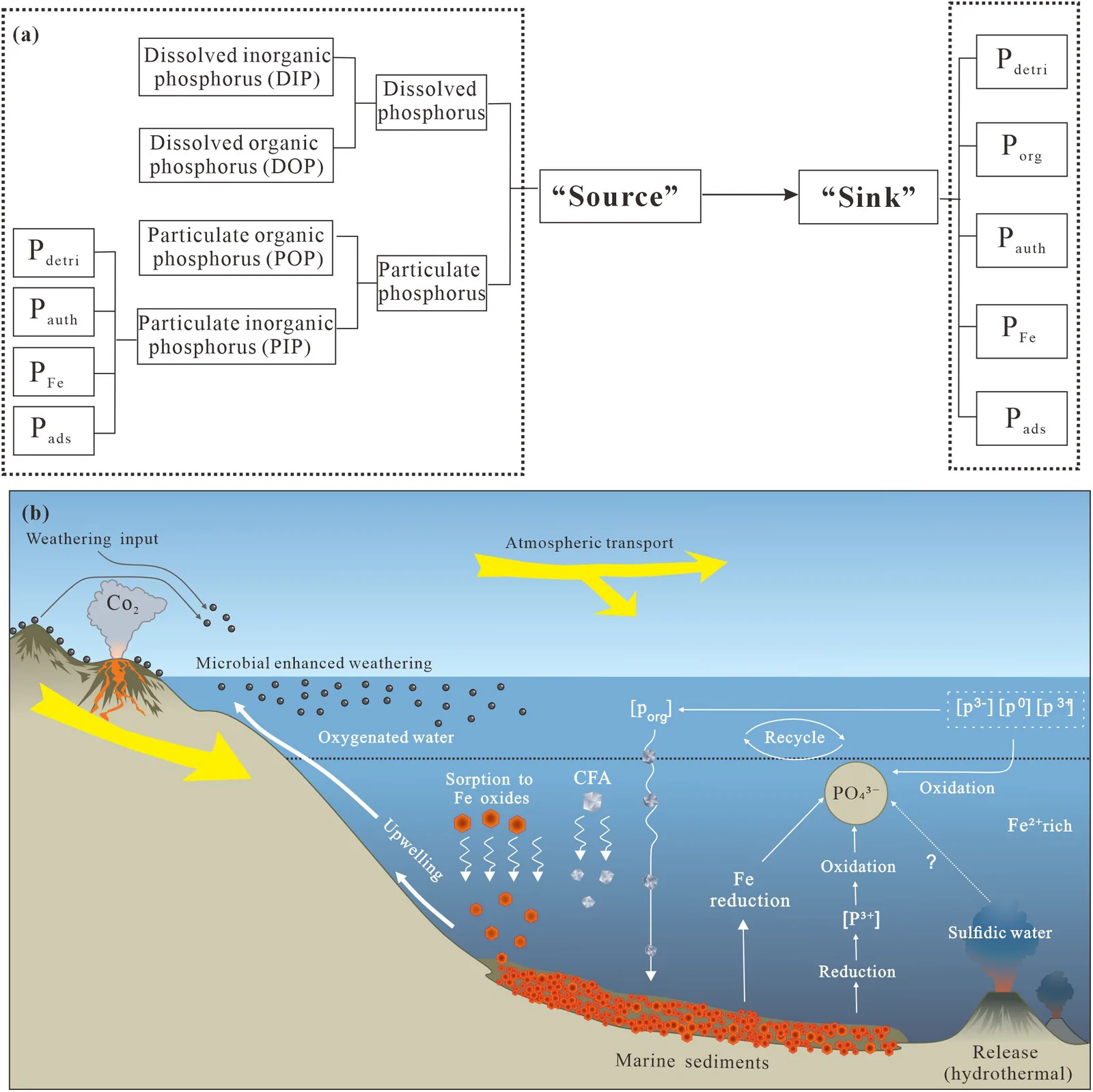
P sources, sinks and recycling pathways. (a) Schematic Diagram of Phosphorus “Source” types, cycling pathways, and phosphorus “Sink” processes. (b) After the origin of life, biological sinks and P recycling pathways, the data was from [1].

Phosphorus in the marine reservoir is supplied primarily by continental weathering input, hydrothermal activity, volcanism, and sedimentary recycling. Among these, the weathering products of continental phosphorus-bearing rocks are the most important source of phosphorus in the ocean, with their flux greatly exceeding that of other sources [5,27]. Recycled phosphorus refers to phosphorus that has been buried and is diffused into seawater through reductive dissolution (phosphorus bound to iron oxides or the remineralization of organic matter. Over geological timescales, hydrothermal and volcanic activities have had a limited impact on marine phosphorus reservoirs compared to continental fluxes and recycling [1,27–29]. Phosphorus from terrestrial input is mainly transported by rivers, which can be divided into two parts: particulate phosphorus (accounting for ~90%) and dissolved phosphorus. Both can be further classified into organic phosphorus and inorganic phosphorus. [28]. Particulate inorganic phosphorus encompasses detrital phosphorus (P_detri_), authigenic phosphorus (P_auth_, CFA), Fe-bound phosphorus (P_Fe_), and adsorbed phosphorus (P_ads_) [5], (Fig 4a-b). Of these, particulate inorganic phosphorus, represented by detrital phosphorus (P_detri_), cannot be directly utilized by marine organisms. Only after riverine phosphorus is converted into reactive phosphorus (dissolved, adsorbed, and organic phosphorus) can it be assimilated by organisms and thereby sustain enhanced primary productivity in the oceans.

Marine phosphorus reservoirs achieve phosphorus burial mainly through two pathways: first, reactive phosphorus is assimilated by organisms and deposited into sed-iments after their death, or microbial activity (e.g., sulfur bacteria) drive phosphogenesis [30–32]. second, phosphorus is adsorbed by iron (hydr) oxide particles and preserved in sediments as Fe-bound phosphorus [5]. However, when organic matter is oxidized or Fe-bound phosphorus is reduced, large amounts of phosphorus can be released from sediments. The decomposition of organic matter increases porewater alkalinity, leading to the precipitation of phosphorus as authigenic apatite (carbonate fluorapatite) within sediments, while the remaining phosphorus diffuses upward into the marine phosphorus reservoir [9]. Therefore, the phosphorus ultimately buried in sediments (total phosphorus, P_total_) mainly comprises organic phosphorus, Fe-bound phosphorus, authigenic phospho-rus, and detrital phosphorus.

#### 5.1.2. Continental weathering and controlling factors during the E-Ꞓ transition.

During the billion years from the late Paleoproterozoic to the early Neoproterozoic (1.8-0.8 Ga), the atmospheric oxygen concentrations were less than 1% of the present atmospheric level, and the oceans remained in a long-term anoxic state, with biological evolution stagnating [33]. This period, characterized by extremely low organic carbon burial efficiency, is commonly referred to as the “Boring Billion”. Previous studies suggested that anoxic and sulfidic oceans lacked key trace elements such as Mo, Cu and Ba required for biological nitrogen fixation, which acted as limiting factors [34]. However, recent research has shown that sulfidation of less than 10% of seafloor area is sufficient to remove Mo from seawater, and that the oceans were more likely characterized by iron-rich seawater [9,35]. This condition is adequate to support biological nitrogen fixation. Therefore, prior to the Neoproterozoic, the primary limiting factor on marine primary productivity and organic carbon burial is more likely phosphorus rather than nitrogen. In the sedimentary record of the Cryogenian Period, the phosphorus content and phosphorite abundance in-creased sharply [36], showing a temporal coupling with the Neoproterozoic Oxidation Event, the appearance of the Ediacaran biota, and the widespread deposition of sedimentary phosphorites in the Ediacaran Period [37–39].

**Fig 5:**
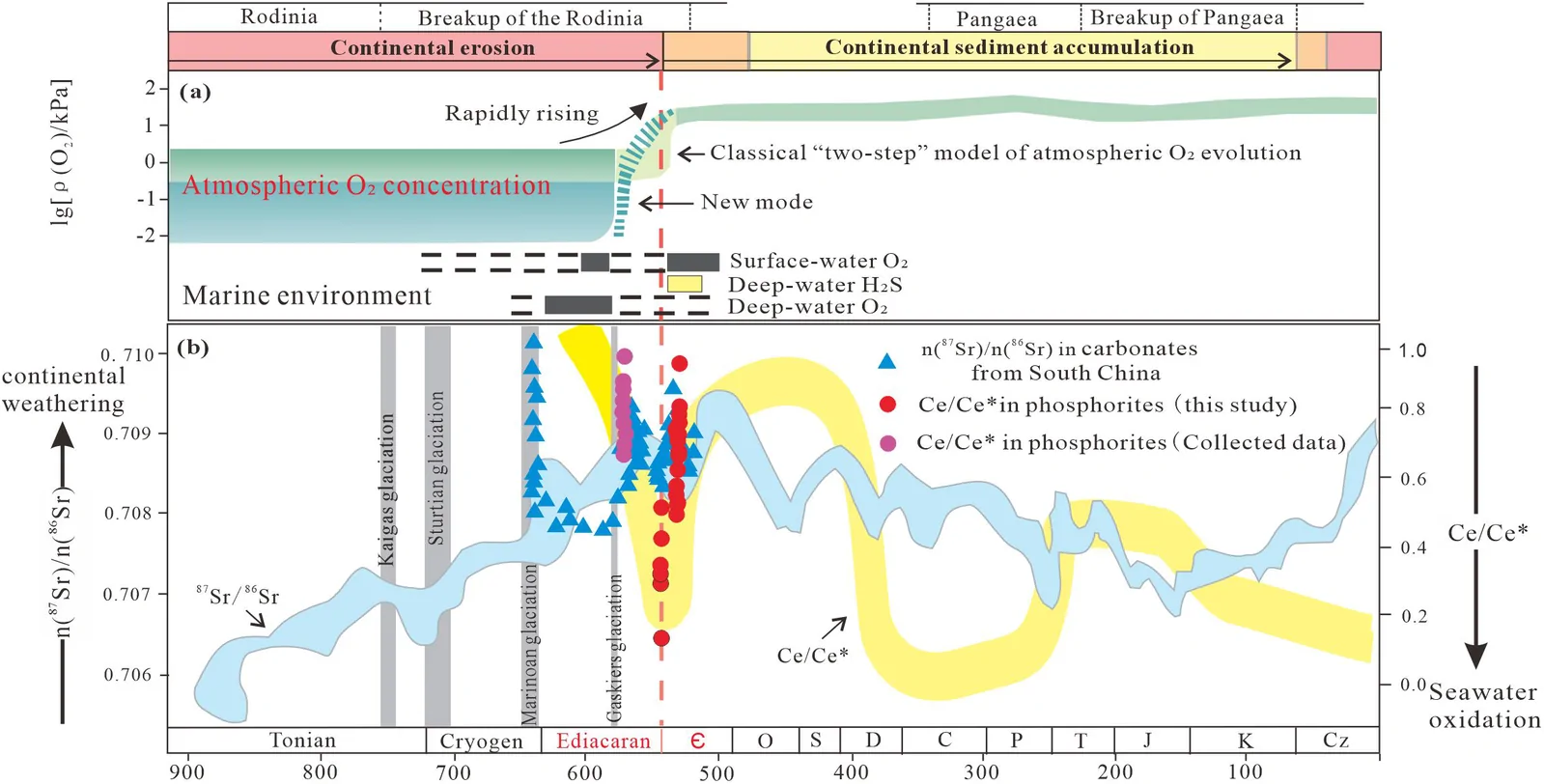
Tectonics, oxygen concentration, and continental weathering since the Neoproterozoic. (a) Variations in atmospheric and oceanic oxygen concentrations; (b) Intensity of continental weathering and redox state of seawater; data of Ce/Ce* was from [8] and this study, and n(^87^Sr) /n(^86^Sr) was from [7,17,18].

**Fig 6:**
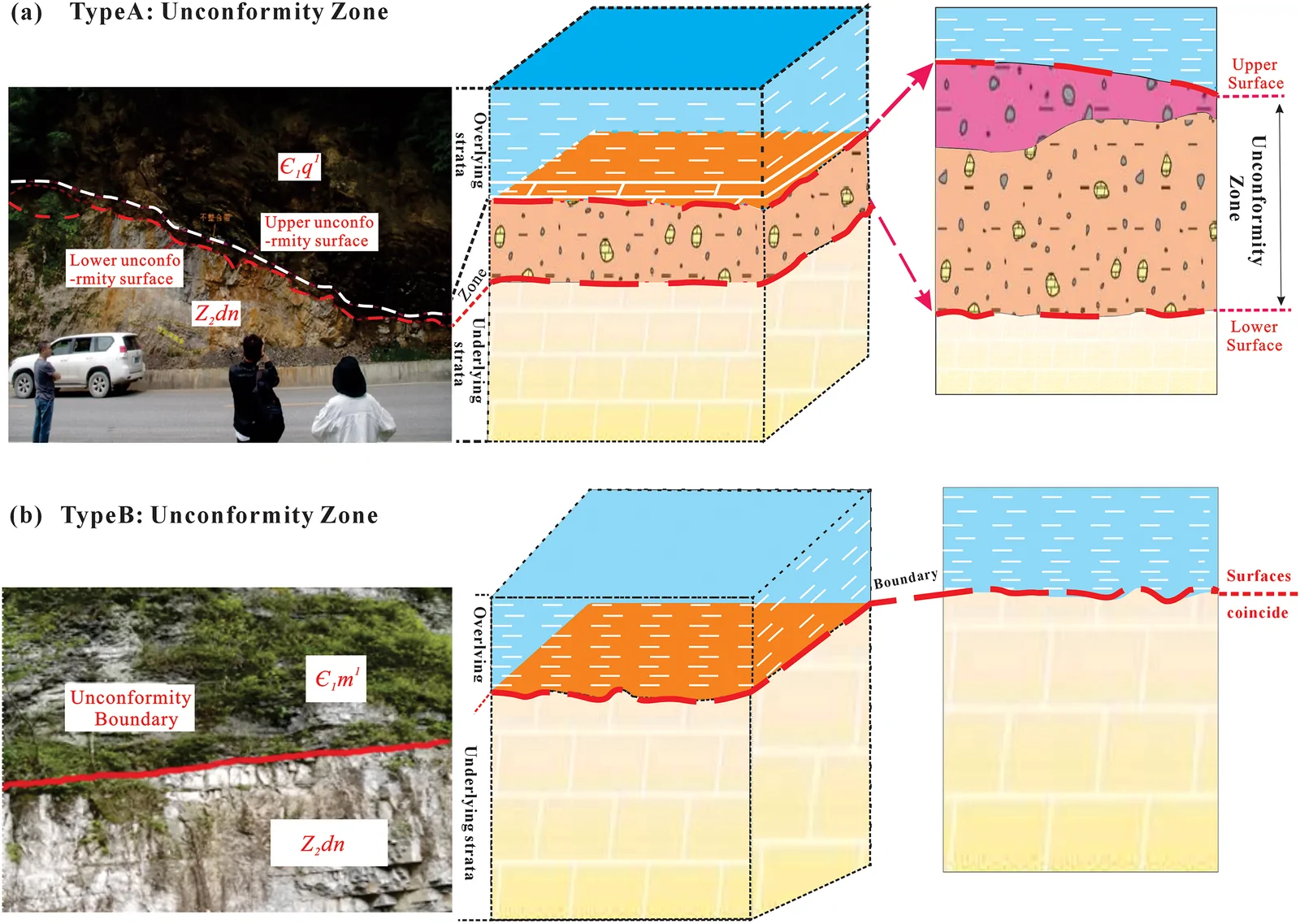
Schematic diagram of structural features in the Late Ediacaran to Early Cambrian unconformity zone on the western margin of the Yangtze Plate.

As illustrated in Fig 5, the late Precambrian was marked by pronounced climatic fluctuations. Following the termination of the Gaskiers glaciation, Earth entered a warm interval during which atmospheric O_2_ levels rose sharply, triggering intense chemical weathering of Precambrian strata. The Ce anomalies preserved in carbonate rocks provide further evidence of atmospheric oxygenation [8], Ce^3+^  was oxidized to Ce⁴⁺ and precipitat-ed as CeO_2_, generating negative Ce anomalies in oxygen-rich seawater [40,41]. The average Ce/Ce* ratio declined from ~0.8 in the Ediacaran Period to ~0.4 in the Cambrian Period, implying substantially higher atmospheric oxygen concentrations during the Cambrian relative to that in the Precambrian (Fig 5). Elevated temperatures and rising oxygen concentrations promoted widespread marine transgressions and basement modification in the Early Cambrian. These processes led to the development of the globally recognized “Great Unconformity,” marked by the Cambrian strata unconformably overlying the Ediacaran succession [42,43]. This unconformity defines the Ediacaran–Cambrian boundary, and two types of unconformity are observed on the southwestern margin of the Yangtze Plate [6]. One form is that the unconformity zone has a certain thickness (Type A), with the upper and lower boundaries corresponding to the lower and upper unconformity lines respectively. The other form is that the unconformity zone has no thickness in space (Type B), and the upper and lower unconformity lines overlap to form a fluctuating boundary line (Fig 6). Along the north-northwest-trending Mianyang-Changning extensional trough in the western margin of the Yangtze Plate, Type A unconformities predominate, expressed by the contact between silt-bearing mudstone of the Lower Cambrian Qiongzhusi Formation and dolostone of the Dengying Formation. Within the extensional trough or on the gentle slopes of the platform margin, Type B unconformities are more common, where phosphatic siliceous mudstone of the Lower Cambrian Maidiping For-mation rests unconformably on dolostone of the Dengying Formation. Only in deep-water basin settings does the Ediacaran-Cambrian contact remain conformable.

Tectonic uplift enhances the exposure of fresh rock surfaces, with accelerated erosion increasing the total weathering flux. In contrast, inactive tectonic activity reduces terrigenous phosphorus delivery and has been regarded as a primary driver of the prolonged low productivity and evolutionary stasis that characterized the “Boring Billion” [9]. During the late Ediacaran–Early Cambrian, the western margin of the Yangtze Plate was profoundly shaped by the three stages of the Tongwan Movement. Each stage followed a consistent evolutionary process of “uplift and erosion first, then subsidence and filling.” There stages correspond to the stratigraphic boundaries between Member III and Member II of the Dengying Formation, between the Maidiping Formation and Dengying Formation, and between the Qiongzhusi Formation and Maidiping Formation. Owing to partial overlap between uplift-erosion and subsidence domains, the Maidiping/Dengying and Qiongzhusi/Maidiping unconformities exhibit regional continuity, forming a near north-south zonal distribution in the western margin of the Sichuan Basin [10]. These unconformity zones reflect episodes of intense erosion and an increase in total weathering flux.

It is noteworthy that the chemical weathering proxies α^Al^Na and α^Al^K exhibit nearly identical trends and vary synchronously with P_2_O_5_ concentrations in the TCG section. Both proxies show elevated values in the upper Dengying Formation and Members 1–2 of the Maidiping Formation, followed by a marked decline in Member 3 of the Maidiping Formation (Fig 3). The marked increase in ^87^Sr/^86^Sr ratios beginning in the late Ediacaran likewise signals intensified continental weathering during the Early Cambrian (ore-forming period) (Fig 5). According to the classical “Uplift-Weathering Hypothesis,” on timescales exceeding one million years, tec-tonic uplift augments erosion fluxes and accelerates continental weathering, with the rapid drawdown of atmospheric CO_2_ exerting major impacts on land-ocean material cycling and climate changes [44–47].

In summary, we have briefly outlined the processes from phosphorus sources to sinks. Although the transformation processes between different phosphorus components in the ocean are not yet fully clear [29]. overall, the flux of total phosphorus from continental weathering to burial is mainly controlled by the combined effects of tectonic activity and climate change [32]. First, the degree of tectonic activity influences the rate of rock erosion, thereby determining the total phosphorus flux. Second, the intensity of chemical weathering affects the proportion of reactive phosphorus within the total flux. Finally, the burial rate of phosphorus is jointly determined by terrigenous input and marine environmental conditions.

### 5.2 Marine redox conditions during the Meishucunian age of the Early Cambrian

The Meishucunian Age of the Early Cambrian marks a critical interval of phospho-genesis in the Yangtze Plate, where the marine redox environment has long been recognized as a major control on phosphorite formation [18,20,21,48]. Concentrations of re-dox-sensitive trace elements (e.g., Ce, U, and Mo) in sedimentary rocks are commonly employed as reliable proxies for marine redox environment. However, existing studies have indicated that individual elemental proxies often show interpretative ambiguity and inherent limitations [26]. U and Mo are enriched in sediments via diffusion under reducing conditions, both reflecting the redox state of bottom waters: uranium enrichment typically denotes anoxic environments, whereas strong molybdenum enrichment is indicative of sulfidic (H_2_S-bearing) bottom waters. The classical Ce anomaly also carries interpretive constraints. In oxygenated seawater, Ce^3+^  is oxidized to Ce⁴⁺ and precipitates as insoluble CeO_2_, causing its decoupling from La and Pr and generating a negative Ce anomaly [49].

**Fig 7:**
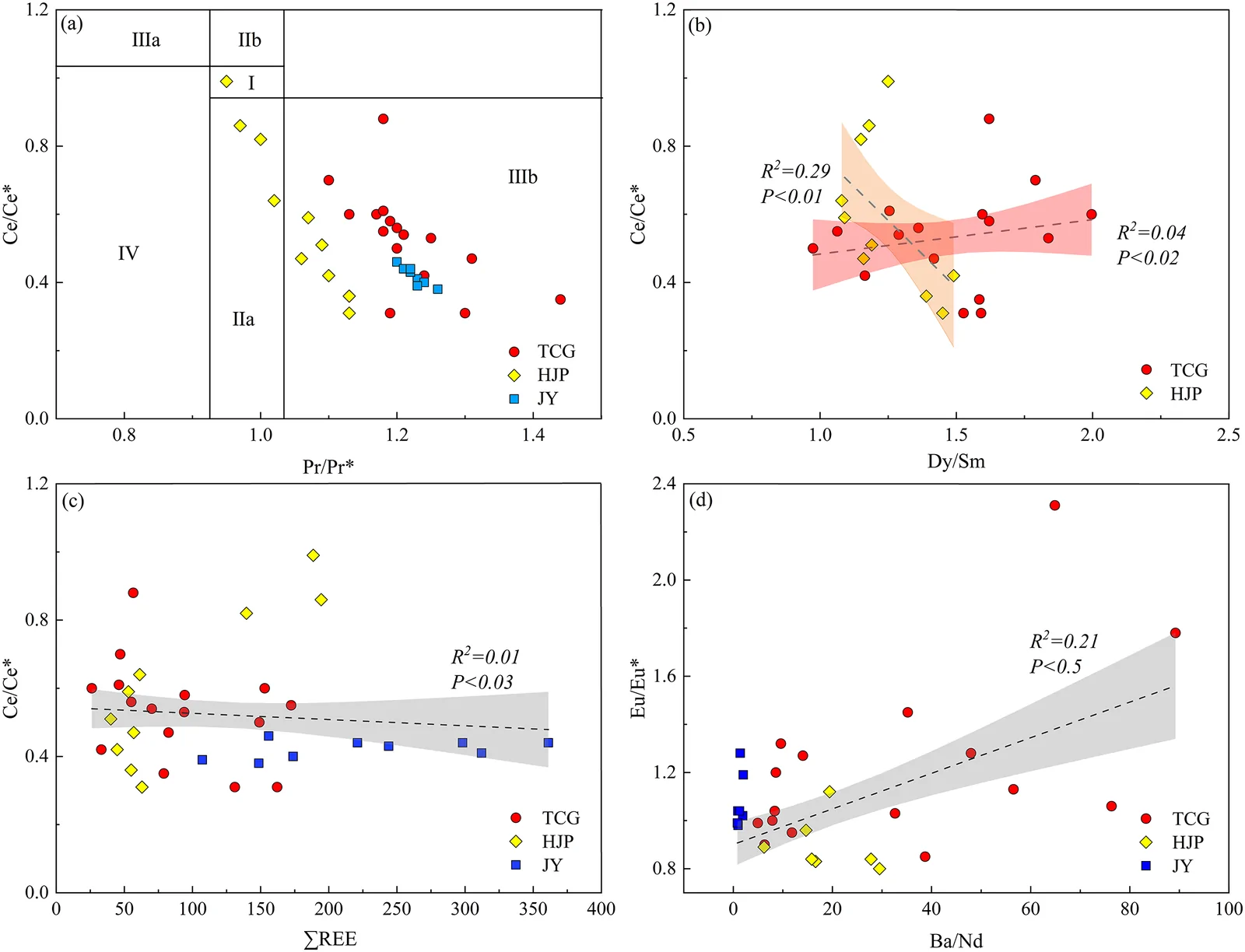
Relationships between Ce/Ce, Eu/Eu, and selected REE parameters in sedimentary rocks from the study area. (a) Ce/Ce* versus Pr/Pr* diagram [25]; (b) Ce/Ce* versus Dy/Sm; (c) Ce/Ce* versus ΣREE; (d) Eu/Eu* versus Ba/Nd. The geochemical parameters are presented in Table 2. Ⅰ, no anomaly; Ⅱa, apparent negative Ce anomaly caused by a positive La anomaly; Ⅱb, apparent positive Ce anomaly caused by a negative La anomaly; Ⅲa, true positive Ce anomaly; Ⅲb, true negative Ce anomaly; Ⅳ, apparent positive Ce anomaly caused by a positive La anomaly.

Studies of the modern ocean reveal that shallow waters (<200 m) exhibit pronounced negative Ce anomalies, reflecting the average oxidation state of seawater, which is strongly depth-dependent. Surface waters are markedly influenced by atmos-pheric O_2_ concentrations, whereas anoxic bottom waters typically display weaker negative Ce anomalies [8]. Compared with Pr and Nd, enrichment of La may signally exaggerate the magnitude of negative Ce anomalies. Therefore, the potential influence of La enrichment should be evaluated prior to interpreting Ce anomalies. The Ce/Ce*-Pr/Pr* discrimination diagram provides an effective approach for distinguishing true Ce anomalies from those induced by La anomalies [25]. As shown in Fig 7a, most samples from the three sections plot within field IIIb, indicating genuine negative Ce anomalies, whereas only four samples from the HJP section fall near the boundary between fields I and IIa. Furthermore, diagenetic alteration of phosphorites is commonly accompanied by enrichment of REEs and a marked decrease in DyN/SmN ratios, resulting in a negative correlation between Ce/Ce* and Dy_N_/Sm_N_ and a positive correlation between Ce/Ce* and ΣREE [20]. However, Ce/Ce* shows no significant correlation with Dy_N_/Sm_N_ (R^2^ < 0.30, P < 0.02) or ΣREE (R^2^ = 0.01, P < 0.03) (Fig 7b-c). These results suggest that diagenetic alteration exerted only a limited influence on the REE compositions, and that the observed Ce anomalies primarily represent primary seawater signatures. Consequently, Ce/Ce* can be regarded as a reliable proxy for reconstructing paleo-redox conditions.

**Table 2:** Selected REE(10^−6^) Concentrations and REE Geochemical Parameters from TCG and HJP.

Sample	La	Sm	Gd	Dy	Yb	Eu/Eu*	Ce/Ce*	Pr/Pr*	La/Yb	La/Sm	Ba/Nd	Dy/Sm	∑REE
TCG01	0.84	1.58	1.76	1.84	0.55	1.20	0.42	1.24	1.52	0.53	8.59	1.16	33.14
TCG02	3.54	5.18	6.66	8.24	2.84	1.27	0.31	1.19	1.25	0.68	14.04	1.59	131.09
TCG04	3.48	3.02	4.07	5.55	2.30	2.31	0.53	1.25	1.51	1.15	64.88	1.84	93.93
TCG05−2	5.22	6.32	7.14	6.72	1.84	1.32	0.55	1.18	2.84	0.83	9.60	1.06	172.42
TCG06	5.15	4.62	5.26	4.50	1.28	1.28	0.50	1.20	4.02	1.11	47.96	0.97	149.12
TCG07−2	3.98	5.70	6.14	8.70	4.79	1.03	0.31	1.30	0.83	0.70	32.63	1.53	162.15
TCG08	1.58	1.95	2.30	3.16	1.48	1.78	0.88	1.18	1.07	0.81	89.22	1.62	56.53
TCG10	2.13	3.47	3.79	4.92	1.97	1.45	0.47	1.31	1.08	0.61	35.20	1.42	82.47
TCG12	4.94	5.61	7.62	11.20	5.05	0.85	0.60	1.17	0.98	0.88	38.71	2.00	153.00
TCG14	2.27	2.84	3.44	4.50	1.96	1.06	0.35	1.44	1.16	0.80	76.32	1.58	78.96
TCG15	0.67	0.89	1.08	1.42	0.79	1.13	0.60	1.13	0.85	0.75	56.54	1.60	26.13
TCG16	1.04	1.62	1.92	2.90	1.67	0.95	0.70	1.10	0.62	0.64	11.83	1.79	46.99
TCG17	2.28	3.40	3.95	5.51	3.40	0.90	0.58	1.19	0.67	0.67	6.31	1.62	94.27
TCG20	1.80	2.70	2.92	3.48	1.48	1.00	0.54	1.21	1.22	0.67	7.88	1.29	70.11
TCG22	1.48	1.94	2.11	2.64	1.20	0.99	0.56	1.20	1.23	0.76	4.94	1.36	55.08
TCG24	1.19	1.61	1.63	2.02	1.14	1.04	0.61	1.18	1.04	0.74	8.36	1.25	46.02
HJP01	17.00	2.27	2.66	2.22	0.78	0.89	0.47	1.06	1.61	1.09	6.21	1.16	56.92
HJP02	32.30	5.15	5.28	4.99	2.44	0.54	0.82	1.00	0.98	0.91	8.06	1.15	139.60
HJP03	34.90	7.46	7.23	7.88	5.73	0.26	0.99	0.95	0.45	0.68	11.24	1.25	188.66
HJP04	17.70	2.17	2.70	2.55	1.16	0.84	0.36	1.13	1.13	1.19	27.79	1.39	55.02
HJP05	22.40	2.34	3.11	2.86	1.14	1.12	0.31	1.13	1.45	1.39	19.44	1.45	62.97
HJP06	13.90	2.16	2.28	1.99	0.80	0.83	0.59	1.07	1.28	0.93	16.61	1.09	53.03
HJP07	16.90	2.28	2.46	2.08	0.87	0.84	0.64	1.02	1.43	1.08	15.83	1.08	61.30
HJP08	44.50	7.11	7.05	7.06	4.08	0.49	0.86	0.97	0.80	0.91	18.78	1.18	194.45
HJP09	13.50	1.70	2.20	2.14	1.02	0.80	0.42	1.10	0.98	1.15	29.52	1.49	44.79
HJP10	11.50	1.55	1.80	1.56	0.63	0.96	0.51	1.09	1.35	1.08	14.67	1.19	40.10

According to the geochemical data from the TCG section, redox proxies (Ce/Ce*, U, and Mo) and paleo-productivity indicators (TOC) show limited variations in Members I and III of the Maidiping Formation, whereas substantial fluctuations are ob-served in Member II (the phosphorite-bearing interval). The ore-bearing interval can be divided into three distinct units.

Unit 1: During the earliest Early Cambrian, rising atmospheric O_2_ concentrations (Fig 6) corresponded with a progressive decrease in Ce/Ce* from ~0.6 to ~0.1 (Fig 3), reflecting increasingly oxygenated surface seawater conditions. In contrast, concurrent enrichments of U_auth_ and Mo_auth_ indicate that bottom waters remained strongly anoxic. Although enhanced atmospheric oxygen levels could have promoted nutrient availability and the preservation of organic matter under reducing conditions, primary productivity remained low during the early Cambrian, resulting in TOC values staying at a low level (TOC < 1%). It is worth noting that Eu, as a variable-valence REE, is not preferentially released under anoxic conditions compared with other REEs. Therefore, the positive Eu anomalies observed in phosphorites are interpreted to reflect contributions from potential hydrothermal fluids or volcanic material input. This inference is supported by the incompatibility of Eu^2+^ within the apatite crystal lattice and by the characteristic positive Eu anomalies in modern hydrothermal deposits [21,48]. In this study, Eu/Eu shows no significant correlation with Ba/Nd (R^2^ = 0.21, P > 0.05; Fig 7d), indicating that the measured Eu anomalies were not significantly affected by elevated Ba contents, and that the observed Eu anomalies are likely genuine The decoupling between Ba and TOC also suggests that the elevated Ba contents in the sediments may reflect the combined influence of potential seafloor hydrothermal activity and anoxic bottom-water conditions. In this case, Ba does not indicate the content of authigenic barite, nor can it reflect the level of marine biological productivity. Furthermore, the participation of sulfur bacteria in the mineralization process cannot be ruled out. Studies of shelf sediments of Namibia and Peru have shown that under anoxic conditions, sulfur bacteria can release sufficient phosphate to trigger the precipitation of hydroxylapatite [30,31].

**Fig 8:**
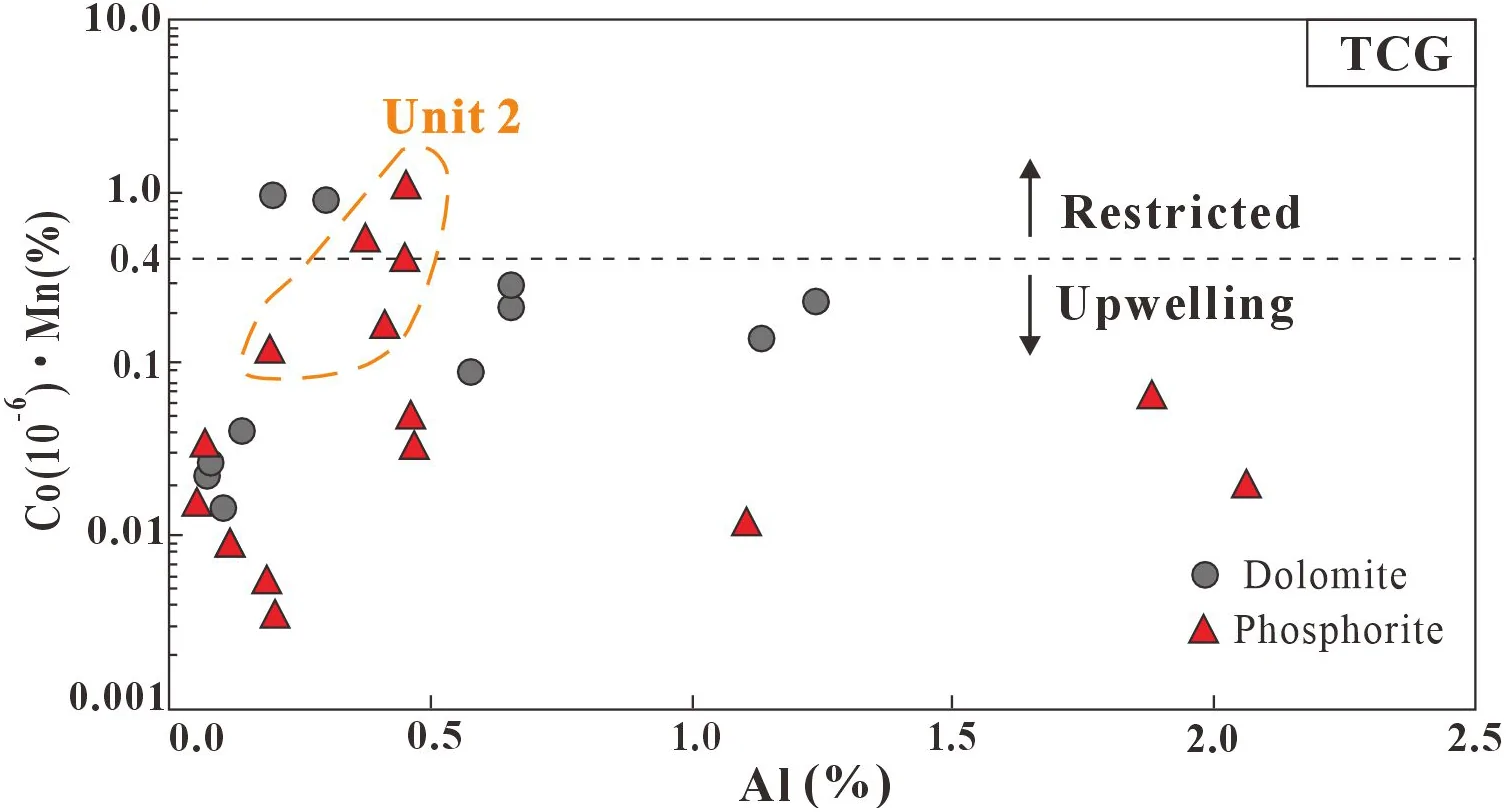
Bivariate plots of Co (10^−6^). Mn (%) against Al (%) in TCG sediments [49]. The geochemical parameters are presented in S4 Table.

Unit 2: This unit exhibits characteristics distinct from those of Unit 1. Ce/Ce* values fluctuate markedly (with a minimum of ~0.3 and a maximum of ~1.0), with an average of ~0.65, reflecting that the average surface seawaters became more anoxic, which provided favorable conditions for the preservation of organic matter. In contrast, U_auth,_ Mo_auth_, and Ba contents are markedly lower than in those in Unit 1, and no significant Eu anomaly is observed, indicating that hydrothermal activity exerted a diminished influence and the activity of upwelling may be restricted during this unit (Fig 8). The concomitant in-crease in TOC content (2.7%−3.7%) suggests a rapid enhancement of terrestrial input, elevated surface-water productivity, or improved organic matter preservation.

Unit 3: In this unit, Ce/Ce* remained relatively stable (with a mean value < 0.7), while U_auth_ concentrations increased to 60 × 10^−6^ and Mo_auth_ levels showed little variation. These geochemical features suggest that both surface and bottom waters were persistently oxygen-depleted, yet bottom waters did not become sulfidic. Sustained oxygen deficiency likely reduced the supply of nutrient-rich detritus from terrigenous input, thereby sup-pressing primary productivity and keeping TOC at low levels. No significant Eu/Eu* anomaly was observed, whereas Ba concentrations increased sharply, probably attributed to enhanced upwelling that introduced oxygen-depleted bottom waters and Ba-enriched sediments into the depositional system.

In summary, the ore-bearing succession of the Maidiping Formation during the Meishucunian Age documents a three-phase evolution of marine redox dynamics and productivity. Unit 1 was marked by oxidized surface waters and strongly reducing, locally euxinic bottom waters, coupled with low primary productivity. Positive Eu anomalies and Ba enrichment may reflect the influence of potential hydrothermal activity. Unit 2 reflects a transition of surface waters toward suboxic conditions, accompanied by reduced hydrothermal input and a significant rise in TOC (2.7%−3.7%), indicating a rapid enhancement in terrestrial nutrient supply, marine primary productivity, and/or organic matter preservation efficiency. The geochemical data in Unit 3 indicate persistent hypoxia in both surface and bottom waters, limiting nutrient supply and suppressing primary productivity, resulting in low TOC accumulation. The sharp Ba increase without Eu anomaly suggests enhanced upwelling of anoxic, Ba-rich bottom waters.

### 5.3 Phosphorus burial model on the western margin of the Yangtze Plate

Marine carbonate sedimentation was the dominant sedimentary type on the Yangtze Plate during the Meishucunian Age of the Early Cambrian [11–13]. Well-developed paleocontinents and island chains surrounded the plate’s southwestern and northern margins, while relatively depressed belts existed between the paleo-uplifts [14]. Controlled by north-south trending paleo-faults, these depressed belts formed large phospho-rite-depositing basins that were geologically favorable for phosphorus accumulation [43,48]. The Maidiping Formation was deposited during a renewed marine transgression that followed regression of the late Ediacaran Dengying Formation. Climatic warming intensified continental weathering, giving rise to the “Great Unconformity” between the Dengying Formation and Maidiping Formation. Rivers at this time delivered substantial phosphorus fluxes to the oceans, which, together with hydrothermal and volcanic inputs, facilitated a major marine phosphorus reservoir.

**Fig 9:**
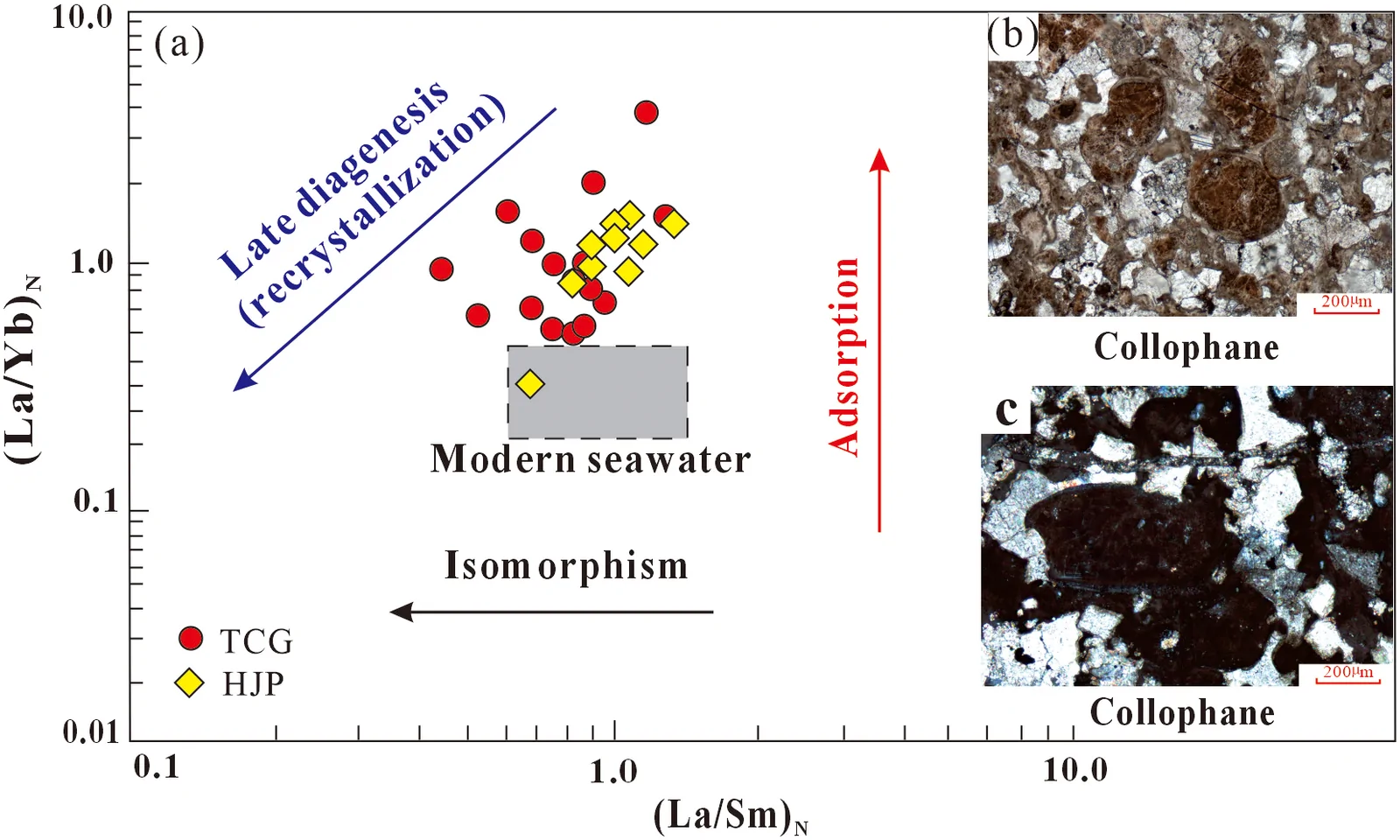
Variations of (La/Yb)_N_ with (La/Sm)_N_ ratios in TCG sediments [46]. The geochemical parameters are presented in Table 2 and S5 Table.

Research on fossil bioapatite indicates that REE distribution patterns record the combined influence of environmental conditions and biological processes on seawater composition through geological time. The La/Yb ratio is largely insensitive to isomorphic substitution but tends to increase when adsorption processes dominate. In contrast, the La/Sm ratio is only weakly affected by adsorption yet can rise markedly through substitution [50]. Additionally, La/Yb and La/Sm ratios in seawaters typically range from 0.2 to 0.5 and 0.6 to 1.6, respectively. Compared with continental freshwater, seawater exhibits a narrower range of variation in these ratios, whereas estuarine waters generally have La/Yb and La/Sm ratios that fall between those of the two [50]. Consequently, besides the direct deposition of detrital phosphate, authigenic collophane formed through phosphate adsorption also constitutes one of the dominant forms of phosphorus burial on the seafloor (Fig 9). Geochemical data are presented in Table 2.

**Fig 10:**
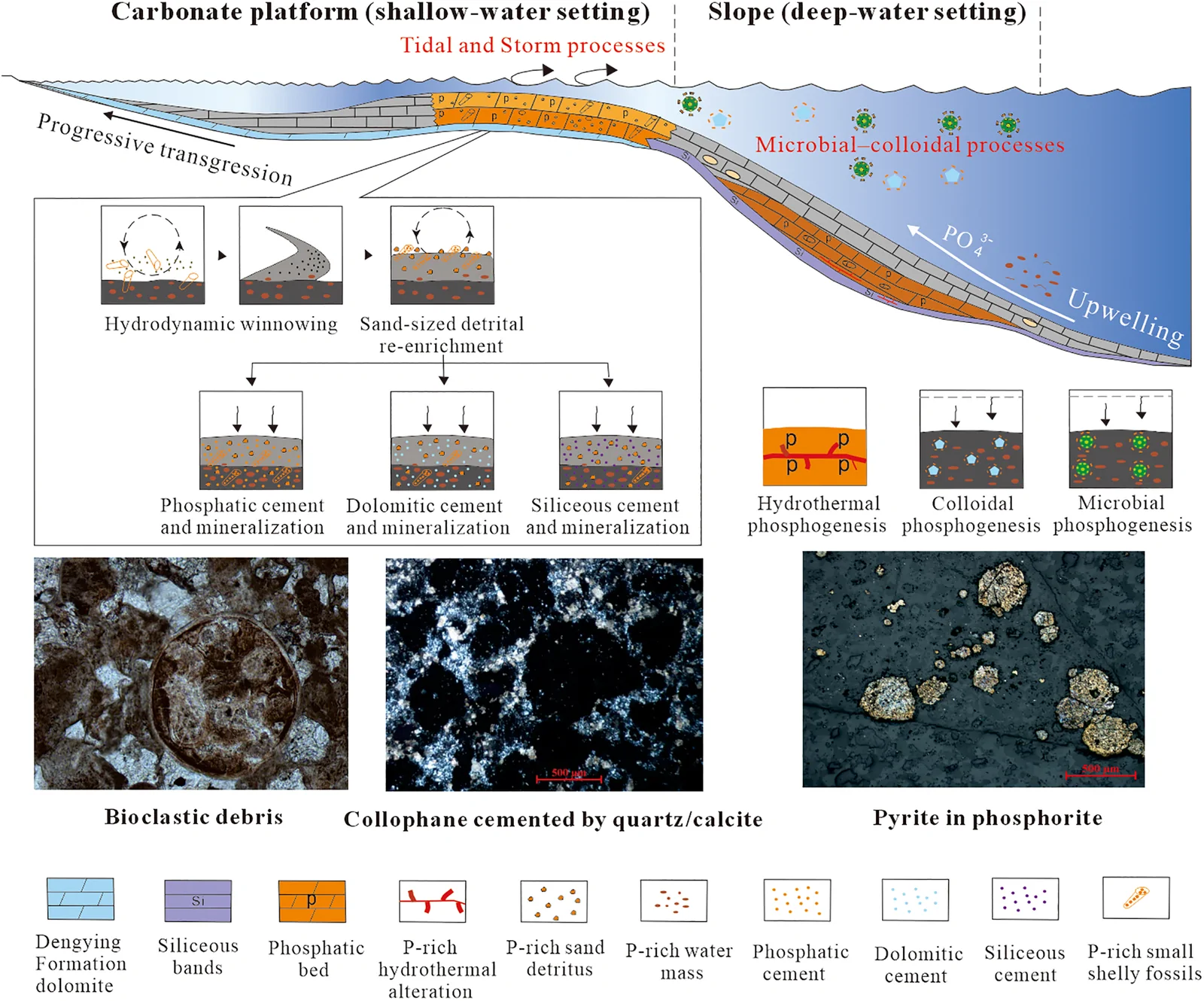
Schematic diagram of phosphorus enrichment and mineralization models in the Early Cambrian of the western margin of the Yangtze Plate.

With ongoing sea-level rise and strengthened marine transgressions, upwelling transported deep anoxic seawaters or hydrothermal inputs to the vicinity of platform-margin slopes [32]. Under favorable geological conditions, sedimentation occurred, leading to distinct positive Eu anomalies and enrichments in Ba, U_auth_, and Mo_auth_ in the sedimentary rocks [51–55]. Contemporaneously, biological activity was limited, whereas the occurrence of abundant pyrite (Fig 10) and siliceous bands suggests that sulfur bacteria and potential hydrothermal processes may have contributed to phosphogenesis [26–31,51–55]. During Unit 2, the influence of upwelling and hydrothermal activity waned considerably, and biological phosphorus accumulation appears to have been enhanced. This phase is marked by elevated TOC values, abundant microbial fossils within phosphatic blasts, and the formation of collophane [54–59]. By Unit 3, renewed transgression expanded phosphorus-rich upwelling into shallow-marine areas such as platforms and tidal flats. Driven by external forces, such as tides, waves, and storms, the high-energy, dynamic oxic environment facilitated the winnowing and secondary enrichment of phosphorus, resulting in the deposition of extensive medium- to thick-bedded conglomeratic phosphorites. Accordingly, the mechanisms of phosphorus enrichment varied distinctly across these units [60]. Overall, the ore-forming materials were sourced primarily from intensified continental weathering, which delivered enhanced phosphorus fluxes to the oceans, supplemented by hydrothermal inputs. Through the transport by upwelling and the mediation of biological processes, these materials provided the fundamental substance for the eventual formation of phosphorites, which were then concentrated into ore bodies under suitable depositional environments. These source-to-sink dynamics of phosphorus governed the total pool of bioavailable phosphorus in the oceans, regulated surface primary productivity, controlled organic carbon generation and burial fluxes, and ultimately exerted pro-found feedbacks on the redox state of the global ocean-atmosphere system and on climate change.

## 6. Conclusions

The marine phosphorus reservoir was supplied predominantly by terrigenous inputs, supplemented by hydrothermal activity and recycled phosphorus. The flux of phosphorus from continental weathering to ultimate burial was jointly regulated by tectonic processes and climate change. Tectonic activity governed rock erosion rates and thus the magnitude of total phosphorus flux, whereas chemical weathering intensity determined the relative proportion of reactive phosphorus. The combined influence of terrigenous inputs and marine environmental conditions ultimately controlled phosphorus burial.

Prior to the Neoproterozoic, phosphorus rather than nitrogen was likely the principal limiting factor for marine primary productivity and organic carbon burial. At the Ediacaran-Cambrian transition, the rapid rise in atmospheric O_2_ concentrations enhanced continental weathering and increased phosphorus delivery to the oceans, resulting in the establishment of a large marine phosphorus reservoir that provided favorable conditions for Early Cambrian phosphogenesis on the Yangtze Plate.

Phosphorus enrichment along the western margin of the Yangtze Plate during the Early Cambrian was controlled by different mechanisms at different depositional stages. Upwelling served as the primary control on phosphogenesis and was accompanied by repeated fluctuations in marine redox conditions. Secondary processes, including biologically mediated phosphorus accumulation, mechanical winnowing, and potential hydrothermal activity, further promoted phosphorus enrichment, collectively resulting in a stage-dependent and multi-process phosphogenic system.

## Supporting information

S1 Table TOC, P_2_O_5_, Ce/Ce*, Eu/Eu*, α^Al^Na and α^Al^K from TCG. (XLSX)

S2 Table Ba, U_auth_ and Mo_auth_ from TCG. (XLSX)

S3 Table Comparison of U_auth_, Mo_auth_, and (Mo/U)_auth_ Parameters for Sediments in the TCG Section and Black Shale. (XLSX)

S4 Table Co (10^−6^)·Mn (%) vs Al (%) in TCG. (XLSX)

S5 Table Selected REE(10^−6^) Concentrations and REE Geochemical Parameters from TCG and HJP. (XLSX)
